# FFBSKAT: Fast Family-Based Sequence Kernel Association Test

**DOI:** 10.1371/journal.pone.0099407

**Published:** 2014-06-06

**Authors:** Gulnara R. Svishcheva, Nadezhda M. Belonogova, Tatiana I. Axenovich

**Affiliations:** 1 Institute of Cytology and Genetics, Siberian Branch of the Russian Academy of Sciences, Novosibirsk, Russia; 2 Novosibirsk State University, Novosibirsk, Russia; University of North Carolina, United States of America

## Abstract

The kernel machine-based regression is an efficient approach to region-based association analysis aimed at identification of rare genetic variants. However, this method is computationally complex. The running time of kernel-based association analysis becomes especially long for samples with genetic (sub) structures, thus increasing the need to develop new and effective methods, algorithms, and software packages. We have developed a new R-package called fast family-based sequence kernel association test (FFBSKAT) for analysis of quantitative traits in samples of related individuals. This software implements a score-based variance component test to assess the association of a given set of single nucleotide polymorphisms with a continuous phenotype. We compared the performance of our software with that of two existing software for family-based sequence kernel association testing, namely, ASKAT and famSKAT, using the Genetic Analysis Workshop 17 family sample. Results demonstrate that FFBSKAT is several times faster than other available programs. In addition, the calculations of the three-compared software were similarly accurate. With respect to the available analysis modes, we combined the advantages of both ASKAT and famSKAT and added new options to empower FFBSKAT users. The FFBSKAT package is fast, user-friendly, and provides an easy-to-use method to perform whole-exome kernel machine-based regression association analysis of quantitative traits in samples of related individuals. The FFBSKAT package, along with its manual, is available for free download at http://mga.bionet.nsc.ru/soft/FFBSKAT/.

## Introduction

The development of new and effective whole-exome and whole-genome resequencing technologies demands the establishment of powerful and computationally efficient statistical methods to test the associations between rare variants and complex traits. Methods developed for the analysis of common variants can be used to map rare variants; however, these methods are underpowered because of the small number of observations for any given variant and the more stringent multiple-test correction compared with that for common variants [1], [2]. The statistical power of the association analysis of rare variants is expected to increase when genetic variants in a region of interest are tested simultaneously instead of separately [1], [2]. The simultaneous consideration of a set of variants from a gene or metabolic pathway not only increases the number of observations for a set of rare variants and decreases the number of tests but also simplifies the interpretation of results [3].

The simplest approach to region-based association analysis uses various methods for collapsing rare variants within a region of interest. In this case, a set of rare variants in a region is replaced by a single genetic variable that is then tested for association through conventional genome-wide association study (GWAS) methods [1], [4]–[6]. Therefore, the computational complexity of regional association analysis based on the collapsing approach is similar to that of GWAS, where fast software packages have been developed even for structured samples (e.g., [7]–[10]). However, the power of association analysis based on the collapsing approach decreases when numerous rare variants are not causal or the effects of causal variants have opposite directions [11].

An alternative approach that employs kernel machine regression has been proposed for regional association analysis [12]–[16]. With respect to quantitative traits, this method compares the average similarity of a set of single nucleotide polymorphisms (SNPs) in the analyzed region for each pair of individuals with pairwise phenotypic similarities. Pairwise genetic similarity is measured by using a kernel function, which reduces the information on multiple SNPs for a pair of individuals into a single scalar factor. Compared with collapsing-based methods, kernel-based methods are more robust to the effects of causal variants with opposite directions, the limited number of causal variants, and the “lower MAF, larger effect size” assumption [16]–[18]. A number of software programs have been developed to conduct kernel-based association tests [16], [17], [19]; an example is the sequence kernel association test (SKAT) [16], which is commonly used to analyze independent samples. The use of this software to approximately analyze related samples after special phenotype transformation has been suggested [20].

A method that involves the use of kernel machine regression has been extended to genetically related samples by three independent scientific groups [21]–[23]. This method provides a score-based variance component test to assess the association of a given SNP set with a continuous phenotype using the restricted likelihood approach. Two software, namely, adjusted SKAT (ASKAT) [22] and family-based SKAT (famSKAT) [23], implement this method. However, the running times of these software programs are long when the sample size and/or the number of regions are large. Therefore, new and effective algorithms and software packages must be developed.

In this study, we propose novel software called fast family-based SKAT (FFBSKAT), which is faster and offers more available analysis modes compared with ASKAT and famSKAT.

## Method and Implementation

In the framework of the kernel machine approach, the inheritance of a quantitative trait in the sample of *n* genetically related individuals is described by the linear mixed model as ***y*** ** ***=*** ** ***Xα*** ** ***+*** ** ***h*** ** ***+*** ** ***b*** ** ***+*** ** ***ε***, where ***y*** denotes the *n*×1 vector of phenotypes; ***X*** represents the *n*×*p* matrix of covariates; ***α*** is the *p*×1 vector of the regression coefficients of the covariates; and ***h***, ***b***, and ***ε*** are the *n*×1 vectors of random effects. As in Ref. [21], we assume that ***h*** is normally distributed with a mean of 0 and covariance *τ* ***K***, *N*(0, *τ* ***K***), where ***K*** denotes the *n*×*n* matrix with elements defined by the kernel function of individual genotypes in the analyzed region, and *τ* denotes the variance component representing the correlations resulting from regional genotypes. For the weighted linear kernel function, ***K*** *=* ***GWWG*** *^T^*, where ***G*** denotes the *n*×*m* matrix of individual genotypes in the analyzed region, and ***W*** represents the *m*×*m* diagonal matrix of SNP weights. Vector ***b*** is assumed to be distributed as *N*(0, *σ_b_* ^2^ ***R***), where ***R*** is the *n*×*n* relationship (twice kinship) matrix, and *σ_b_* ^2^ is the variance component that models within-family correlations; ***ε***∼*N*(0, *σ_e_* ^2^ ***I***_n_), where ***I***_n_ is the *n*×*n* identity matrix, and *σ_e_* ^2^ is the variance component of random errors. In this model, the quantitative trait follows a multivariate normal distribution with the vector of means ***Xα*** and the covariance matrix *σ_b_* ^2^ ***R*** *+ τ* ***K*** + *σ_e_* ^2^ ***I***_n_.

In fitting the null hypothesis (H_0_: *τ* = 0), the variance components *σ_b_* ^2^ and *σ_e_* ^2^ are numerically estimated to calculate the covariance matrix ***V*** = *σ_b_* ^2^ ***R*** + *σ_e_* ^2^ ***I***_n_. Coefficients ***α*** are calculated as ***α*** ** ***= *** (***X*** *^T^* ***V*** ^−1^ ***X***)^−1^ ***X*** *^T^* ***V*** ^−1^ ***y***. The score statistic of *τ* for testing H_0_ is
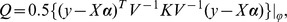
where *φ* denotes the vector of the maximum likelihood estimates of the parameters ***α***, *σ_b_* ^2^, and *σ_e_* ^2^ under H_0_. This score statistic can be rewritten using the projection matrix ***P*** = ***I***_n_ *–* ***X***(***X*** *^T^* ***V*** ^−1^ ***X***)^−1^ ***X*** *^T^* ***V*** ^−1^, that is,



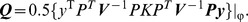



Under H_0_, the score statistic ***Q*** is distributed as Σ*λ_i_χ_i_* ^2^, where ***λ*** is a set of eigenvalues of a matrix 0.5***V*** ^−1/2^ ***PKP*** *^T^* ***V*** ^−1/2^, and *χ_i_* ^2^ is a chi-squared distribution with one degree of freedom [21], [23]. *P* value can be computed analytically using either Davies’ method [24] or Kuonen’s saddlepoint technique [25].

Thus, the kernel-based association analysis of quantitative traits in related samples that uses the variance component score test consists of two steps: estimation of the set of mixed model parameters under H_0_ and calculation of score statistic ***Q*** and the set of eigenvalues ***λ*** for each analyzed region. The first step of the kernel-based association analysis is similar to that of the score-based GWAS methods that are widely used on related samples. A considerable number of efficient algorithms, such as those described by Lippert *et al*. [8] and Svishcheva *et al.* [9], have been developed for this step. Our FFBSKAT software uses the “polygenic” procedure in the GenABEL package v 1.7–2 or later (http://www.genabel.org/for the GenABEL project web-site) for the first step of analysis. Although this analysis step is computationally intensive, it is performed only once for an analyzed trait. The second step is repeated many times for each genomic region and may therefore be a limiting factor in the whole-exome association analysis. We accelerate this step using the following analytical and algorithmic improvements.

1. Invariant matrix operations, whose operands are independent on regional genotypes, are conducted once before the second step of analysis, in which score statistics ***Q*** and *P* values are calculated for the set of analyzed regions (for instance, the matrix operation ***V*** ^−1^ ***Py***).
2. Identical matrix multiplication operations are replaced by the resultant matrix (for example, the operation ***V*** ^−1^ ***X***).
3. For the linear kernel, the *n*×*n* matrix ***V*** ^−1/2^ ***PKP*** *^T^* ***V*** ^−1/2^ is replaced by the *m*×*m* matrix ***WG*** *^T^* ***P*** *^T^* ***V*** ^−1^ ***PGW*** with identical non-null eigenvalues, where the number of SNPs in region *m* is smaller than sample size *n.*
4. Effective algorithms are utilized for linear algebra operations (in the case of nonlinear kernel, we use the Cholesky decomposition to find *V* ^−1/2^ in the expression ***V*** ^−1/2^ ***PKP*** *^T^* ***V*** ^−1/2^, because this method provides the best compromise in terms of both performance and execution time [26]).

We implemented these optimizations in the R-package FFBSKAT. The software programs for the Linux and Windows operating systems are distributed under the GPLv3 license and are available for free on http://mga.bionet.nsc.ru/soft/FFBSKAT/.

## Results and Discussion

We tested the running time of FFBSKAT against ASKAT and famSKAT on the GAW17****mini-exome family dataset [27]. A subset of 500 related individuals in 7 pedigrees genotyped at 9,868 SNPs in 1,550 gene regions was doubled to generate sample sizes of 1,000, 2,000, and 4,000. SNPs were not filtered by MAF, and frequencies varied from 0.001 to 0.5 (mean 0.058, median 0.012). The number of SNPs in a gene region varied from 2 to 102 (mean 6.4, median 4). We used the kinship matrix based on the pedigree structure to avoid the problems arising from the duplication of individuals. Different samples consisted of different numbers of pedigrees (7, 14, 28, and 56 for sample sizes of 500, 1000, 2000, and 4000, respectively), and kinship matrices included 7, 14, 28, and 56 independent blocks. Although the kinship matrices had a block diagonal structure, we did not simplify them during the association analysis and considered it as a full *n*×*n* matrix. We analyzed the Q1 simulated trait. This trait was affected by age, smoking, and 39 SNPs in 9 genes, and it had a residual heritability of 0.44 [27]. Five causal genes with more than one polymorphic SNP were present in our selected sample. These causal SNPs had frequencies ranging from 0.001 to 0.027.

Directly comparing the running time for the analysis of the mini-exome data using FFBSKAT, ASKAT, and famSKAT is not logical. FamSKAT is suited to the analysis of a single genome region. Analyzing numerous regions using famSKAT requires repeating the most computationally intensive first step of the analysis (estimation of the set of mixed model parameters under H_0_) many times. ASKAT does not use an external kinship matrix but calculates it within the package. This tendency also increases the full running time. Thus, to compare and estimate the advantage of our software for the second step of the analysis (calculation of score statistic ***Q*** and estimation of *P* value), we implemented the following experiment design. We calculated the kinship matrix using the information about the structure of the pedigrees and adjusted the Q1 simulated trait on covariates (age and smoking) because ASKAT does not allow covariates. Then, we estimated the mixed model parameters under H_0_ and conducted the invariant matrix operations whose operands were independent of regional genotypes. We extracted procedures for the second step of the analysis from each software and excluded the matrix operations whose operands were independent of regional genotypes from famSKAT and ASKAT. We obtained three procedures, namely, “FFBSKAT”, “ASKAT”, and “famSKAT”, to calculate the score statistic ***Q*** and the *P* value for a single genome region. We sequentially calculated the score statistic ***Q*** and the *P* value for each region of the mini-exome using these procedures and estimated the running time for each procedure (one processor on the computer server that was equipped with 96 GB memory and two Six Core Xeon X5675 3.07 GHz, CentOS release 6.5 [Final] Linux 2.6.32-431.5.1.el6.x86_64).

**Figure 1:**
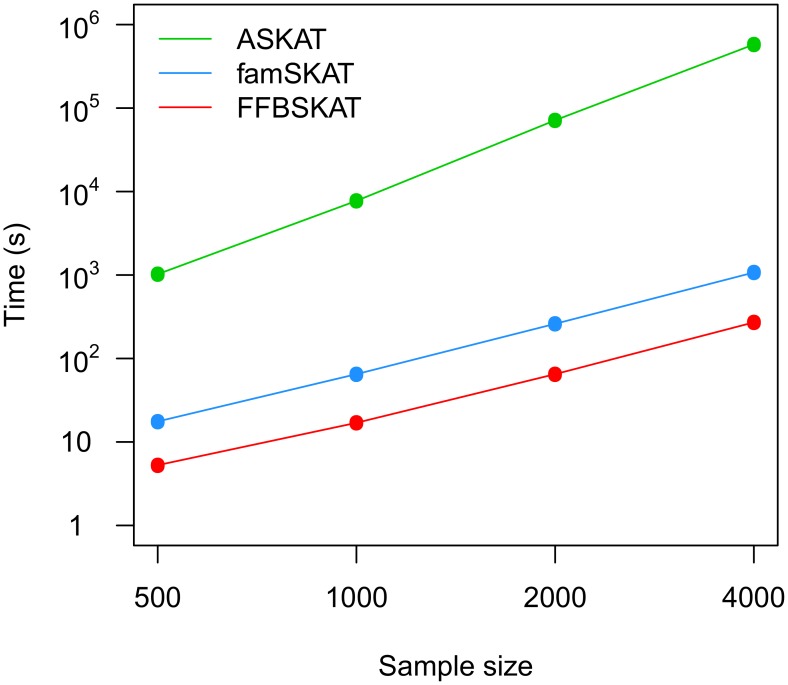
Dependence of the running times of the second step of mini-exome analysis of quantitative trait Q1 on sample size for different methods (using one processor at 3.07 GHz). Points show the estimated running times (*RT*), lines correspond to the linear regression equations: *RT*_ASKAT_ = 9×10^−6^ *n* ^3^–.753; *RT*_famSKAT_ = 6.7×10^–5^ *n* ^2^–2.8, and *RT*_FFBSKAT_ = 1.7×10^–5^ *n* ^2^–3.7, where *n* is the sample size.

The results presented in Fig. 1 show that “ASKAT” displays cubic dependence on sample size, whereas “famSKAT” and “FFBSKAT” exhibit quadratic dependence. “FFBSKAT” is approximately four times faster than “famSKAT”. In our experiment, the “famSKAT” and “ASKAT” procedures were additionally optimized by excluding matrix operations whose operands were independent of genotypes operations. Therefore, the performance of the famSKAT and ASKAT software is expected to be considerably slow in practice.

Directly introducing covariates into the analysis instead of preliminary adjusting for them did not significantly change the running time of “FFBSKAT”. The mean difference between these running times was estimated at −0.004 s on 200 realizations using a sample size of 500 and was not significant (*P*_paired *T*-test_ = 0.199).

Although the aim of our experiment was to estimate the running time for the second step of the analysis, we also estimated the total running time of both steps. The proportion of the first step was as small as 0.7 to 6% of the total running time in whole-exome region-based association analysis of the sample of 500 to 4000 individuals. This result emphasizes practical importance of the acceleration of the second stage.

**Figure 2:**
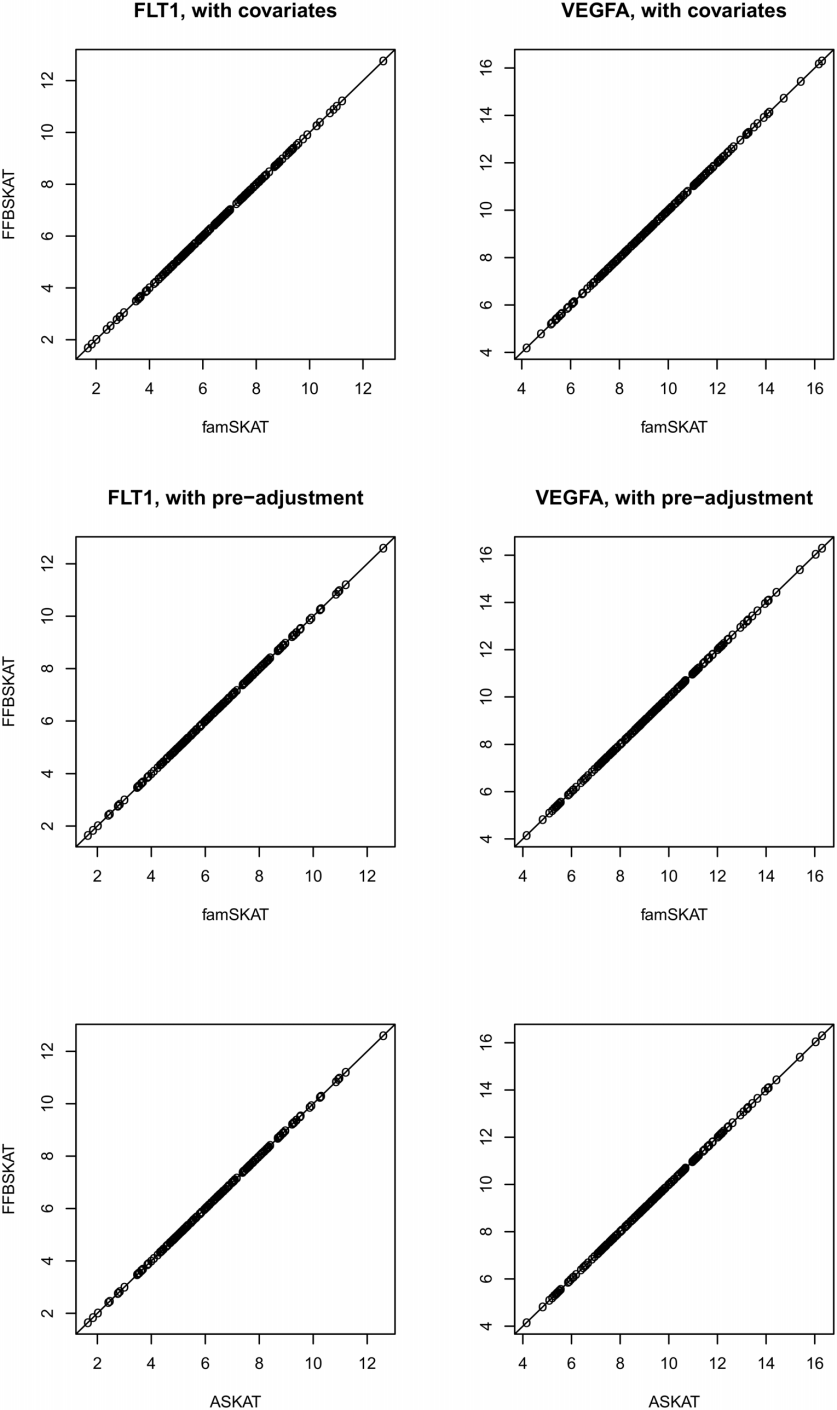
Comparison of the *P* values (shown as minus base 10 logarithm) computed with famSKAT, ASKAT, and FFBSKAT given a sample of 500 individuals, for two causal genes, *FLT1* and *VEGFA*. 200 realizations of Q1 quantitative trait in GAW17 data were analyzed. The line indicates one-to-one correspondence.

For two genes, *VEGFA* and *FLT1*, we compared the *P* values determined by using “FFBSKAT” with those values obtained by using “famSKAT” and “ASKAT” to estimate the accuracy of our software. The *P* values had a clear one-to-one correspondence (Fig. 2). The statistical properties of the methods implemented in ASKAT and famSKAT have been analyzed previously [22], [23]. The pure coincidence of the *P* values for these software and FFBSKAT warrants the identity of the statistical properties of the methods implemented in all three software.

Directly introducing covariates into the regional analysis resulted in *P* values that were close to those obtained after the preliminary adjustment of a trait. However, a paired comparison of the base 10 logarithms of the *P* values shows that adding covariates in the regional analysis slightly reduced the *P* values (*P*_paired *T*-test_ = 4.6×10^–3^ for *FLT1* and 6.6×10^–10^ for *VEGFA*). Therefore, introducing covariates into regional analysis yields a slightly higher power than a simple pre-adjustment does; that is, at α = 5×10^–8^, the power changed from 0.320 to 0.325 for *FLT1* and from 0.810 to 0.825 for *VEGFA*.

Aside from being the fastest method for conducting kernel-based association tests for quantitative traits in related samples, FFBSKAT offers the most extensive list of analysis options. FFBSKAT allows the assignment of different kernel functions (not only linear but also polynomial, IBS-based etc.) and arbitrary weight functions and supports covariates and non-additive models. It can also analyze a set of regions in either parallel or sequential modes and estimate *P* values using either Davies’ or Kuonen’s methods.

FFBSKAT can utilize a kinship matrix calculated using either the pedigree structure (pedigree kinship) or the genotypes of a large number of common SNPs (genomic kinship), whereas ASKAT and famSKAT use only genomic and pedigree kinship matrices, respectively. A genome-wide association analysis using genomic kinship has consistently been shown to be more powerful than pedigree-based analysis [28]. This effect may be explained by two factors: errors in genealogy can distort the pedigree kinship, and kinship coefficient computed from a pedigree is an expectation of the proportion of the genome shared identically by descent even though the true proportion of the genome shared may deviate from this expectation [29]. In spite of this effect, the use of pedigree kinship may be computationally effective if the sample includes several pedigrees, because in this case the kinship matrix has a block structure. However, we do not simplify the kinship matrix in our software, so using the pedigree-based instead of genome-based kinship matrix does not change essentially the running time. The pedigree kinship is also preferable if the number of SNPs is small. The GAW17 pedigree dataset includes only 3,121 polymorphic SNPs with MAF >5%, thus indicating that adjusting for pedigree-based kinship results in better control for false-positive rate than adjusting for genomic kinship in the region-based association analysis of rare variants based on the collapsing approach [30]. Thus, the possibility of utilizing any type of kinship matrix by FFBSKAT is an important advantage of our software. Therefore, our FFBSKAT package has more advantages than ASKAT and famSKAT do not only in terms of speed but also with respect to its features.
